# A third monoclinic polymorph of 3,4,5-trihy­droxy­benzoic acid monohydrate

**DOI:** 10.1107/S1600536811018848

**Published:** 2011-05-25

**Authors:** Güneş Demirtaş, Necmi Dege, Orhan Büyükgüngör

**Affiliations:** a Department of Physics, Arts and Sciences Faculty, Ondokuz Mayıs University, 55139 Samsun, Turkey

## Abstract

The title compound, C_7_H_6_O_5_·H_2_O, is a new polymorph of the structures reported by Jiang *et al.* (2000 ▶) [*Acta Cryst.* C**56**, 594–595] and Okabe *et al.* (2001 ▶) [*Acta Cryst.* E**57**, o764–o766]. The gallic acid mol­ecule is essentially planar (r.m.s. deviation = 0.550 Å). An intra­molecular O—H⋯O hydrogen bond occurs in the gallic acid mol­ecule, which is linked to the water mol­ecule by a further O—H⋯O hydrogen bond. In the crystal, the components are linked by O—H⋯O hydrogen bonds. The hydrogen-bonding pattern differs from those reported for the previous polymorphs.

## Related literature

For the biological activity of gallic acid, see: Lu *et al.* (2006 ▶); Madlener *et al.* (2007 ▶). For the previously reproted polymorphs, see: Jiang *et al.* (2000 ▶); Okabe *et al.* (2001 ▶). For a related structure, see: Genç *et al.* (2004 ▶).
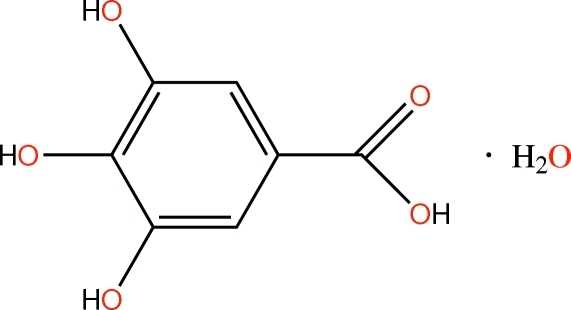


## Experimental

### 

#### Crystal data

C_7_H_6_O_5_·H_2_O
*M* *_r_* = 188.13
Monoclinic, 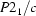

*a* = 9.7943 (7) Å
*b* = 3.6122 (2) Å
*c* = 21.5905 (15) Å
β = 91.268 (6)°
*V* = 763.66 (9) Å^3^
*Z* = 4
Mo *K*α radiation
μ = 0.15 mm^−1^
*T* = 296 K
0.61 × 0.28 × 0.09 mm

#### Data collection

Stoe IPDS 2 diffractometer
Absorption correction: integration (*X-RED32*; Stoe & Cie, 2002 ▶) *T*_min_ = 0.948, *T*_max_ = 0.986
4558 measured reflections
1502 independent reflections
1262 reflections with *I* > 2σ(*I*)
*R*_int_ = 0.042

#### Refinement

*R*[*F* ^2^ > 2σ(*F* ^2^)] = 0.039
*wR*(*F* ^2^) = 0.104
*S* = 1.04
1502 reflections
127 parameters
3 restraints
H atoms treated by a mixture of independent and constrained refinement
Δρ_max_ = 0.15 e Å^−3^
Δρ_min_ = −0.23 e Å^−3^

### 

Data collection: *X-AREA* (Stoe & Cie, 2002 ▶); cell refinement: *X-AREA*; data reduction: *X-RED32* (Stoe & Cie, 2002 ▶); program(s) used to solve structure: *WinGX* (Farrugia, 1997 ▶) and *SHELXS97* (Sheldrick, 2008 ▶); program(s) used to refine structure: *SHELXL97* (Sheldrick, 2008 ▶); molecular graphics: *ORTEP-3 for Windows* (Farrugia, 1997 ▶); software used to prepare material for publication: *WinGX* (Farrugia, 1999 ▶) and *PLATON* (Spek, 2009 ▶).

## Supplementary Material

Crystal structure: contains datablocks I, global. DOI: 10.1107/S1600536811018848/bx2352sup1.cif

Structure factors: contains datablocks I. DOI: 10.1107/S1600536811018848/bx2352Isup2.hkl

Supplementary material file. DOI: 10.1107/S1600536811018848/bx2352Isup3.cml

Additional supplementary materials:  crystallographic information; 3D view; checkCIF report

## Figures and Tables

**Table 1:** Hydrogen-bond geometry (Å, °)

*D*—H⋯*A*	*D*—H	H⋯*A*	*D*⋯*A*	*D*—H⋯*A*
O2—H3⋯O3	0.82	2.26	2.7006 (18)	114
O5—H6⋯O6	0.82	1.85	2.6542 (17)	167
O1—H2⋯O6^i^	0.82	2.03	2.7539 (19)	147
O2—H3⋯O3^ii^	0.82	2.52	3.1721 (19)	137
O3—H4⋯O4^iii^	0.82	1.94	2.7154 (19)	158
O6—H6*A*⋯O2^iv^	0.83 (2)	2.03 (2)	2.8237 (19)	161 (3)
O6—H6*B*⋯O1^v^	0.82 (2)	2.00 (2)	2.814 (2)	171 (5)

Symmetry codes: (i) 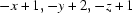; (ii) 
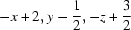
; (iii) 
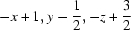
; (iv) 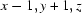; (v) 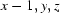.

**Table 2:** Other crystal structures of gallic acid monohydrate (Å, °)

	1	2
Unit-cell parameters	*a* = 5.794 (4)	*a* = 14.15 (1)
	*b* = 4.719 (5)	*b* = 3.622 (9)
	*c* = 28.688 (5)	*c* = 15.028 (10)
	β = 95.08 (3)	β = 97.52 (7)
	*V* = 781.4 (3)	*V* = 764 (1)
Space group	Monoclinic, *P*2_1_/*c*	Monoclinic, *P*2/*n*
Reference	Jiang *et al.* (2000 ▶)	Okabe *et al.* (2001 ▶)

**Fig. 1.**
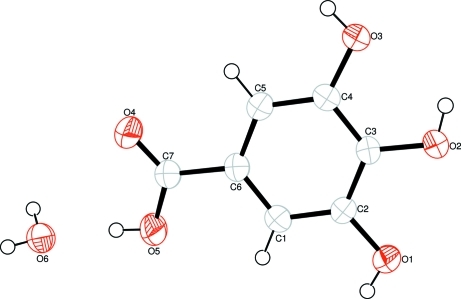
The asymmetric unit of the title compound, showing the atomic numbering scheme. Displacement ellipsoids are drawn at the 50% probability level.

**Fig. 2.**
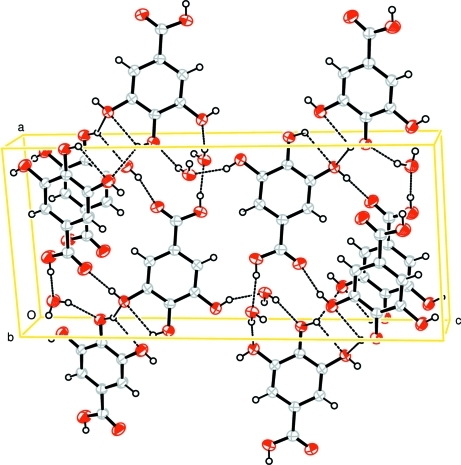
Packing diagram for (I), showing the three-dimensional hydrogen bonding array. Hydrogen bonds are shown as dashed lines.

## supplementary crystallographic information

### Comment

Gallic acid and its derivatives are a group of naturally occurring polyphenol antioxidants which have recently been shown to have potential healty effects as an excellent free radical scavenger (Lu *et al.*, 2006). In adition, gallic acid block DNA synthesis in leukemia cells (Madlener *et al.*, 2007). Up to now, the different crystal structure of gallic acid monohidrate form have been reported by (Jiang *et al.*, 2000; Okabe *et al.*, 2001). The unit-cell parameters belong to these crystal structures have given at Table 2.

We report here the crystal structure of the title compound, C_7_H_6_O_5_.H_2_O. The asymmetric unit of title compound, C_7_H_6_O_5_.H_2_O, consists of one 3,4,5-trihydroxybenzoic acid molecule and one water molecule (Fig. 1). The C—C bond distances range from 1.378 (2) Å to 1.480 (2) Å. The longest C—C bond distance is between C6 and C7 with 1.480 Å. The C—O bond distance are range from 1.212 (2) Å to 1.3723 (19) Å. The shortest C—O bond distance is between C7 and O4 with 1.212 (2) Å. The C—O bond distance for different crystal structure was given 1.359 (2) Å by (Genç *et al.*, 2004).

The crystal structure has two intramolecular hydrogen bonds and five intermolecular hydrogen bonds (Table 1). The O2—H3···O3 and O5—H6···O6 hydrogen bonds present in the asymmetric unit. In the crystal structure, the gallic acid molecules and water molecules are linked by the O6—H6A···O2, O6—H6B···O1, O5—H6···O6 and O1—H2···O6 hydrogen bonds. While the C—H···O hydrogen bond is present in crystal structure reported by (Jiang *et al.*, 2000), there isn't present in our crystal structure. The *via* hydrogen atom of water molecule makes bifurcated hydrogen bond in crystal structure reported by (Jiang *et al.*, 2000),but this kind hydrogen bond is not present in our crystal structure. In crystal structure reported by (Okabe *et al.*, 2001), the hydroxyl groups make two intramolecular hydrogen bonds.

The torsion angles at ring belong to gallic acid molecule are range from 0.0 (3)° to 1.9 (3)°. Therefore, the gallic acid molecule close to planar. The tosion angles for the C2—C1—C6—C7 and C4—C5—C6—C7 are 179.51 (16)° and -179.08 (17)°, respectively. The O4—C7—O5 angle is 122.82 (15)°.

### Experimental

A hot solution (60 °C) of gallic acid (0.002 mol, 0.340 g) in distilled water (approximately 20 ml) was gradually added to a hot stirring solution of magnesium sulfate heptahydrate (MgSO_4_.7H_2_O) (0.001 mol, 0.246 g) in distilled water (approximately 20 ml). The obtained mixture was stirred on a hot plate with slow evaporation to 30 ml at 60° C. The mixture was left for crystallization and in a few days, the crystals were obtained by slow evaporation from the solution at room temperature.

### Refinement

H6A and H6B atoms were located in a difference map and were refined isotropically, with O—H distances in the range 0.830 (18)-0.824 (19) Å, and the H···H distance of 1.320 (2) Å. The other H atoms were positioned geometrically and refined using a riding model, with C—H = 0.930 Å, O—H = 0.820 Å, and *U*_iso_(H) = 1.2*U*_eq_(C) and 1.5*U*_eq_(O).

### Crystal data

**Table:**

C_7_H_6_O_5_·H_2_O	*F*(000) = 392
*M**_r_* = 188.13	*D*_x_ = 1.636 Mg m^−^^3^
Monoclinic, *P*2_1_/*c*	Mo *K*α radiation, λ = 0.71073 Å
Hall symbol: -P 2ybc	Cell parameters from 6472 reflections
*a* = 9.7943 (7) Å	θ = 1.9–27.6°
*b* = 3.6122 (2) Å	µ = 0.15 mm^−^^1^
*c* = 21.5905 (15) Å	*T* = 296 K
β = 91.268 (6)°	Needle, pale brown
*V* = 763.66 (9) Å^3^	0.61 × 0.28 × 0.09 mm
*Z* = 4	

### Data collection

**Table:**

Stoe IPDS 2 diffractometer	1502 independent reflections
Radiation source: fine-focus sealed tube	1262 reflections with *I* > 2σ(*I*)
graphite	*R*_int_ = 0.042
w–scan rotation	θ_max_ = 26.0°, θ_min_ = 1.9°
Absorption correction: integration (*X-RED32*; Stoe & Cie, 2002)	*h* = −9→12
*T*_min_ = 0.948, *T*_max_ = 0.986	*k* = −4→4
4558 measured reflections	*l* = −26→26

### Refinement

**Table:**

Refinement on *F*^2^	Secondary atom site location: difference Fourier map
Least-squares matrix: full	Hydrogen site location: inferred from neighbouring sites
*R*[*F*^2^ > 2σ(*F*^2^)] = 0.039	H atoms treated by a mixture of independent and constrained refinement
*wR*(*F*^2^) = 0.104	*w* = 1/[σ^2^(*F*_o_^2^) + (0.0466*P*)^2^ + 0.2493*P*] where *P* = (*F*_o_^2^ + 2*F*_c_^2^)/3
*S* = 1.04	(Δ/σ)_max_ < 0.001
1502 reflections	Δρ_max_ = 0.15 e Å^−^^3^
127 parameters	Δρ_min_ = −0.23 e Å^−^^3^
3 restraints	Extinction correction: *SHELXL97* (Sheldrick, 2008), Fc^*^=kFc[1+0.001xFc^2^λ^3^/sin(2θ)]^-1/4^
Primary atom site location: structure-invariant direct methods	Extinction coefficient: 0.023 (5)

### Special details

**Table:**

Geometry. All e.s.d.'s (except the e.s.d. in the dihedral angle between two l.s. planes) are estimated using the full covariance matrix. The cell e.s.d.'s are taken into account individually in the estimation of e.s.d.'s in distances, angles and torsion angles; correlations between e.s.d.'s in cell parameters are only used when they are defined by crystal symmetry. An approximate (isotropic) treatment of cell e.s.d.'s is used for estimating e.s.d.'s involving l.s. planes.
Refinement. Refinement of *F*^2^ against ALL reflections. The weighted *R*-factor *wR* and goodness of fit *S* are based on *F*^2^, conventional *R*-factors *R* are based on *F*, with *F* set to zero for negative *F*^2^. The threshold expression of *F*^2^ > σ(*F*^2^) is used only for calculating *R*-factors(gt) *etc*. and is not relevant to the choice of reflections for refinement. *R*-factors based on *F*^2^ are statistically about twice as large as those based on *F*, and *R*- factors based on ALL data will be even larger.

### Fractional atomic coordinates and isotropic or equivalent isotropic displacement parameters (Å^2^)

**Table:**

	*x*	*y*	*z*	*U*_iso_*/*U*_eq_	
C1	0.67337 (16)	0.6332 (5)	0.58435 (8)	0.0369 (4)	
H1	0.6383	0.7058	0.5459	0.044*	
C2	0.81149 (16)	0.5670 (5)	0.59282 (8)	0.0349 (4)	
C3	0.86369 (15)	0.4587 (5)	0.65029 (8)	0.0356 (4)	
C4	0.77763 (17)	0.4238 (5)	0.70009 (8)	0.0395 (4)	
C5	0.63957 (17)	0.4863 (5)	0.69214 (8)	0.0414 (4)	
H5	0.5814	0.4596	0.7253	0.050*	
C6	0.58767 (16)	0.5895 (5)	0.63421 (8)	0.0370 (4)	
C7	0.43925 (16)	0.6603 (5)	0.62739 (8)	0.0408 (4)	
O1	0.90229 (12)	0.5986 (4)	0.54561 (6)	0.0480 (4)	
H2	0.8613	0.6599	0.5137	0.072*	
O2	1.00108 (11)	0.3881 (4)	0.65588 (6)	0.0469 (4)	
H3	1.0200	0.3265	0.6916	0.070*	
O3	0.83973 (12)	0.3290 (5)	0.75521 (6)	0.0546 (4)	
H4	0.7823	0.3169	0.7822	0.082*	
O4	0.35878 (13)	0.5981 (5)	0.66792 (6)	0.0583 (4)	
O5	0.40210 (12)	0.7959 (5)	0.57298 (6)	0.0567 (4)	
H6	0.3193	0.8292	0.5719	0.085*	
O6	0.14365 (13)	1.0125 (5)	0.56257 (7)	0.0499 (4)	
H6A	0.120 (3)	1.147 (8)	0.5916 (12)	0.102 (11)*	
H6B	0.077 (4)	0.879 (14)	0.555 (2)	0.24 (3)*	

### Atomic displacement parameters (Å^2^)

**Table:**

	*U*^11^	*U*^22^	*U*^33^	*U*^12^	*U*^13^	*U*^23^
C1	0.0338 (8)	0.0434 (9)	0.0333 (9)	0.0031 (7)	−0.0022 (7)	0.0016 (7)
C2	0.0310 (8)	0.0418 (9)	0.0321 (9)	0.0009 (7)	0.0036 (6)	0.0008 (7)
C3	0.0264 (7)	0.0457 (9)	0.0346 (9)	0.0013 (7)	−0.0009 (6)	−0.0010 (7)
C4	0.0337 (8)	0.0530 (11)	0.0316 (9)	0.0013 (7)	−0.0021 (6)	0.0037 (8)
C5	0.0322 (8)	0.0572 (11)	0.0349 (9)	0.0039 (7)	0.0042 (7)	0.0038 (8)
C6	0.0299 (8)	0.0444 (9)	0.0367 (9)	0.0041 (7)	0.0002 (7)	−0.0001 (7)
C7	0.0319 (8)	0.0525 (10)	0.0381 (10)	0.0049 (7)	0.0003 (7)	−0.0008 (8)
O1	0.0361 (6)	0.0738 (10)	0.0342 (7)	0.0052 (6)	0.0051 (5)	0.0085 (6)
O2	0.0286 (6)	0.0775 (10)	0.0345 (7)	0.0051 (6)	−0.0026 (5)	0.0035 (6)
O3	0.0334 (6)	0.0984 (12)	0.0321 (7)	0.0070 (7)	−0.0005 (5)	0.0138 (7)
O4	0.0335 (6)	0.0953 (12)	0.0465 (8)	0.0101 (7)	0.0074 (6)	0.0062 (8)
O5	0.0330 (6)	0.0882 (11)	0.0488 (8)	0.0137 (7)	−0.0020 (5)	0.0158 (7)
O6	0.0358 (7)	0.0697 (9)	0.0440 (8)	0.0126 (6)	−0.0016 (5)	0.0016 (7)

### Geometric parameters (Å, °)

**Table:**

C1—C2	1.382 (2)	C5—H5	0.9300
C1—C6	1.388 (2)	C6—C7	1.480 (2)
C1—H1	0.9300	C7—O4	1.212 (2)
C2—O1	1.372 (2)	C7—O5	1.317 (2)
C2—C3	1.388 (2)	O1—H2	0.8200
C3—O2	1.3723 (19)	O2—H3	0.8200
C3—C4	1.387 (2)	O3—H4	0.8200
C4—O3	1.368 (2)	O5—H6	0.8200
C4—C5	1.378 (2)	O6—H6A	0.830 (18)
C5—C6	1.391 (2)	O6—H6B	0.824 (19)
			
C2—C1—C6	118.99 (15)	C4—C5—H5	120.2
C2—C1—H1	120.5	C6—C5—H5	120.2
C6—C1—H1	120.5	C1—C6—C5	120.83 (15)
O1—C2—C1	122.41 (14)	C1—C6—C7	120.83 (15)
O1—C2—C3	117.10 (14)	C5—C6—C7	118.32 (15)
C1—C2—C3	120.48 (15)	O4—C7—O5	122.82 (15)
O2—C3—C4	121.78 (14)	O4—C7—C6	123.32 (16)
O2—C3—C2	118.17 (14)	O5—C7—C6	113.86 (15)
C4—C3—C2	120.05 (14)	C2—O1—H2	109.5
O3—C4—C5	124.44 (16)	C3—O2—H3	109.5
O3—C4—C3	115.54 (14)	C4—O3—H4	109.5
C5—C4—C3	120.01 (15)	C7—O5—H6	109.5
C4—C5—C6	119.61 (16)	H6A—O6—H6B	105 (3)
			
C6—C1—C2—O1	178.97 (16)	O3—C4—C5—C6	178.56 (18)
C6—C1—C2—C3	0.0 (3)	C3—C4—C5—C6	−0.9 (3)
O1—C2—C3—O2	−0.5 (2)	C2—C1—C6—C5	1.0 (3)
C1—C2—C3—O2	178.51 (16)	C2—C1—C6—C7	179.51 (16)
O1—C2—C3—C4	179.56 (16)	C4—C5—C6—C1	−0.5 (3)
C1—C2—C3—C4	−1.4 (3)	C4—C5—C6—C7	−179.08 (17)
O2—C3—C4—O3	2.4 (3)	C1—C6—C7—O4	173.92 (19)
C2—C3—C4—O3	−177.66 (17)	C5—C6—C7—O4	−7.5 (3)
O2—C3—C4—C5	−178.04 (17)	C1—C6—C7—O5	−5.8 (3)
C2—C3—C4—C5	1.9 (3)	C5—C6—C7—O5	172.79 (18)

### Hydrogen-bond geometry (Å, °)

**Table:**

*D*—H···*A*	*D*—H	H···*A*	*D*···*A*	*D*—H···*A*
O2—H3···O3	0.82	2.26	2.7006 (18)	114
O5—H6···O6	0.82	1.85	2.6542 (17)	167
O1—H2···O6^i^	0.82	2.03	2.7539 (19)	147
O2—H3···O3^ii^	0.82	2.52	3.1721 (19)	137
O3—H4···O4^iii^	0.82	1.94	2.7154 (19)	158
O6—H6A···O2^iv^	0.83 (2)	2.03 (2)	2.8237 (19)	161 (3)
O6—H6B···O1^v^	0.82 (2)	2.00 (2)	2.814 (2)	171 (5)

Symmetry codes: (i) −*x*+1, −*y*+2, −*z*+1; (ii) −*x*+2, *y*−1/2, −*z*+3/2; (iii) −*x*+1, *y*−1/2, −*z*+3/2; (iv) *x*−1, *y*+1, *z*; (v) *x*−1, *y*, *z*.

### Table 2 Other crystal structures of gallic acid monohydrate (Å, °)

**Table:**

	1	2
Unit-cell parameters	*a* = 5.794 (4)	*a* = 14.15 (1)
	*b* = 4.719 (5)	*b* = 3.622 (9)
	*c* = 28.688 (5)	*c* = 15.028 (10)
	β = 95.08 (3)	β = 97.52 (7)
	*V* = 781.4 (3)	*V* = 764 (1)
Space group	Monoclinic, P2_1_/c	Monoclinic, P2/n
Reference	Jiang *et al.* (2000)	Okabe *et al.* (2001)
